# Psychogenic polidipsia in a patient with bipolar affective disorder (BAD)

**DOI:** 10.1192/j.eurpsy.2021.1661

**Published:** 2021-08-13

**Authors:** A. Sarafopoulos, D. Antoniadis, V. Karpouza

**Affiliations:** 4th Picu, Psychiatric Hospital of Thessaloniki, Thessaloniki, Greece

**Keywords:** Polidipsia, manía

## Abstract

**Introduction:**

A 49 y.o. male patient was admitted to the male PICU with a manic episode. Upon admission he presented with mood elation, pressured speech, lack of sleep, agitation and polydipsia.

**Objectives:**

To investigate the symptom of psychogenic polydipsia in mental health patients presenting with severe mania.

**Methods:**

The patient was assessed regularly by the psychiatric team consisting of a CT doctor and one General Adult Consultant. Appropriate psychological assessments for mania and laboratory investigations took place. There was a referral to Endocrinology for further investigation of the symptom.

**Results:**

The patient initially scored above 40 in the Young Mania Rating Scale (YMRS), establishing a diagnosis of mania. Upon admission he was treated with Paliperidone 9mg OD and Sodium Valproate 1gr OD. The daily dose of the sodium valproate was increased. Concerning the polydipsia, the investigations by the Endocrinology department indicated the specific weight in urine within normal range. The mental health team proceeded in a cross-titration of Paliperidone to Aripiprazole. On the 10th day since admission the management of the manic symptoms was considered satisfactory. Nevertheless, the polydipsia continued to a certain extend.

**Conclusions:**

After ruling out organic and pharmacological causes of the polydipsia, the mental health team wondered about the cause of the symptom. Further investigation is required in order to clarify whether the polydipsia could actually qualify as a symptom in severe mania.

**Disclosure:**

No significant relationships.

